# Case series and techniques of Descemet’s Stripping Automated Endothelial Keratoplasty for severe bullous keratopathy after birth injury

**DOI:** 10.1186/s12886-015-0094-z

**Published:** 2015-08-06

**Authors:** Akira Kobayashi, Hideaki Yokogawa, Natsuko Mori, Kazuhisa Sugiyama

**Affiliations:** Department of Ophthalmology, Kanazawa University Graduate School of Medical Science, 13-1 Takara-machi, Kanazawa-shi, Ishikawa-ken 920-8641 Japan

## Abstract

**Background:**

To evaluate clinical outcomes of Descemet’s Stripping Automated Endothelial Keratoplasty (DSAEK) for severe bullous keratopathy that develop as a late complication of endothelial injury to the baby during forceps delivery at birth.

**Case presentations:**

Four eyes (four patients; mean age, 51.5 years) with severe bullous keratopathy as a late complication of forceps delivery at birth were enrolled. All patients had amblyopia from childhood due to cloudy cornea. Nontheless, DSAEK was indicated in these patients for the irritation and severe light sensation caused by apparent bullous change of the injured cornea. All patients underwent DSAEK and two patients had simultaneous cataract surgery. Intraoperative and postoperative complications were recorded. Postoperative donor endothelial-cell densities (ECDs) were measured prospectively at six and 12 months and compared with preoperative values. Best corrected visual acuity (BCVA) was measured at 6 and 12 months postoperatively. All cases required corneal epithelial removal; two cases with simultaneous cataract surgeries required lens anterior capsule staining by trypan blue and illumination of the cornea for visualization. There were no cases of graft dislocation or primary graft failure. Mean BCVA improved from 0.06 to 0.15 at 6 months and to 0.38 at 12 months. Postoperative ECD was 2270 cells/mm^2^ (mean loss, 24.4 %) at 6 months and 2130 (mean loss, 29.1 %) at 12 months. Postoperative intraocular pressure elevation was observed in two cases, and a rejection episode occurred in one case at 4 months postoperatively.

**Conclusions:**

In this case series, the clinical outcome of DSAEK for severe bullous keratopathy after forceps delivery was fair with rapid corneal clearance, which was comparable to uncomplicated cases. Cataract and DSAEK surgery was safely performed using techniques including epithelial removal, lens anterior capsule staining and illuminating the cornea, which enabled better visualization of the anterior chamber.

## Background

Descemet’s Stripping Automated Endothelial Keratoplasty (DSAEK) is widely performed as a preferred treatment of endothelial dysfunction [1–5]. DSAEK completely eliminates any surface corneal incisions or sutures, maintains much of the structural integrity of the cornea and induces minimal refractive change, suggesting distinct advantages over standard penetrating keratoplasty (PK) [6, 7]. However, DSAEK requires a completely smooth host corneal rear surface since only air pressure is used for donor attachment. Therefore, endothelial surface irregularity is one of the contraindications of DSAEK.

Forceps delivery is sometimes performed in the course of vaginal childbirth when the mother and/or baby are having difficulties during the pushing stage of labor. In a forceps delivery, a doctor applies forceps, an instrument shaped like a pair of large spoons or salad tongs, to the baby's head to help guide the baby out of the birth canal. One complication of a forceps delivery is that the baby’s Descemet’s membrane can be damaged, which causes late corneal endothelial failure with an irregular endothelial surface [8].

In this report, clinical outcomes and detailed surgical tips of DSAEK (and phacoemulsification of concomitant cataract surgery) for severe bullous keratopathy due to endothelial injury after forceps delivery are presented. Also, this report highlights surgical techniques to enhance visualization of severe bullous change in cases of birth injury with or without simultaneous cataract surgery. This information may be useful for DSAEK not only in cases with corneal birth injury, but also in similar corneal endothelial pathologies with severe bullous keratopathy.

## Case presentations

### Patients

**Fig. 1:**
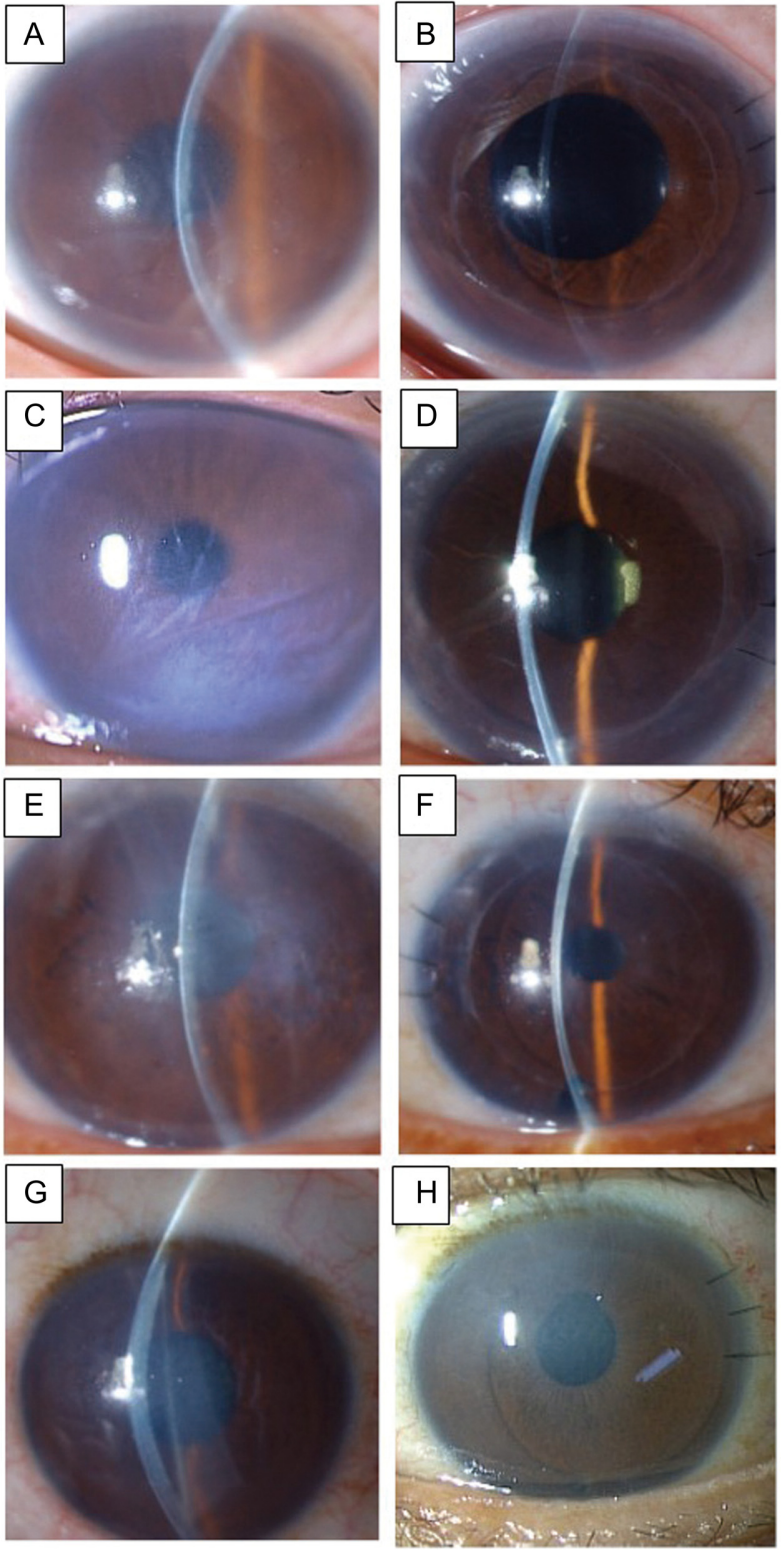
Slit-lamp photos of bullous keratopathies due to endothelial injury after forceps delivery before and after DSAEK. **a** Slit-lamp photo before DSAEK in case 1. A Descemet’s membrane break was observed from the 5-o’clock to the 11-o’clock position. **b** Slit-lamp photo 6 months after DSAEK in case 1. Both the donor and the host corneas were clear with an improvement in visual acuity. **c** Slit-lamp photo before DSAEK in case 2. A Descemet’s membrane break was observed from the 8-o’clock to the 3-o’clock position. A severe bullous change of the cornea was noted. **d** Slit-lamp photo 6 months after DSAEK in case 2. Both the donor and the host corneas were clear with an improvement in visual acuity. **e** Slit-lamp photo before DSAEK in case 3. A Descemet’s membrane break was observed from the 5-o’clock to the 11-o’clock position. A severe bullous change of the cornea was noted. **f** Slit-lamp photo 6 months after DSAEK in case 3. Both the donor and the host corneas were clear with an improvement in visual acuity. **g** Slit-lamp photo before DSAEK in case 4. A Descemet’s membrane break was observed from the 5-o’clock to the 11-o’clock position. **h** Slit-lamp photo 6 months after DSAEK in case 4. Although host stromal haze was still noted, the bullous change had disappeared

This prospective, non-comparative study was approved by the Ethical Committee of Kanazawa University Graduate School of Medical Science and followed the tenets of the Declaration of Helsinki. Written informed consent was obtained from all patients for publication of this Case report and any accompanying images. Four eyes (four patients; mean age, 51.5 years) with bullous keratopathy as a late complication of endothelial injury after forceps delivery at birth were enrolled (Table 1, Fig. 1). All patients had amblyopia from childhood due to cloudy cornea. Therefore, the diagnosis of endothelial injury due to forceps delivery was already made at childhood by characteristic Descemet’s membrane breaks (Haab’s striae). Nontheless, DSAEK was indicated in these patients later in their lives for the irritation and severe light sensation caused by apparent bullous change of the injured cornea.

**Table 1:** Demographic data and clinical outcomes for four patients with bullous keratopathy after forceps delivery

Case	Sex/Age (yrs)	Eye	Surgery	Clinical Diagnosis	Initial BCVA	BCVA 6 months postop/12 months postop	Endothelial cell density (/mm^2^), Preop/6 months postop/12 months postop	Complications	Visualization technique used
1	F/46	OD	DSAEK	BK after forceps delivery	0.15	0.5 /0.6	3088/2583 (16.4 % decrease)/2702 (12.5 % decrease)	None	Epithelial removal
2	F/51	OD	DSAEK	BK after forceps delivery	0.4	0.3 /0.6	2941/2673(9.1 % decrease) /2583(12.2 % decrease)	None	Epithelial removal
3	M/56	OD	DSAEK + PEA + IOL	BK after forceps delivery	0.05	0.15/0.15	2983/1912 (35.9 % decrease)/2352(21.2 % decrease)	Postoperative IOP elevation (12 months postop)	Epithelial removal, rypan blue staining, illumination by an endoillumination probe
4	F/53	OS	DSAEK + PEA + IOL	BK after forceps delivery	Hand motion	0.02/0.02	3000/1912 (36.3 % decrease)/881(70.6 % decrease)	Postoperative IOP elevation (day 1), rejection (4 months postop)	Epithelial removal, trypan blue staining, illumination by a surgical slit lamp

*Abbreviations*: BCVA, best corrected visual acuity; DSAEK, Descemet’s stripping automated endothelial keratoplasty; PEA, phacoemulsification and aspiration; IOL, intraocular lens implantation; BK, bullous keratopathy; IOP, intraocular pressure; N/A, not applicable

### Clinical outcomes

All patients underwent DSAEK; two patients underwent simultaneous cataract surgery. Intraoperative and postoperative complications, including iatrogenic primary graft failure, graft dislocation, and pupillary block glaucoma were documented in all four eyes. Postoperative central donor endothelial-cell densities (ECDs) were measured prospectively at 6 and 12 months and compared with preoperative values. Best corrected visual acuity (BCVA) was measured at 6 and 12 months postoperatively. Decimal visual acuity was used as a measure of visual acuity. Central ECD was measured by noncontact specular microscopy (Nonconrobo, Konan Medical Inc., Hyogo, Japan), using the center method as outlined by the manufacturer’s software. Postoperative cell loss was calculated as a percentage of the preoperative donor ECD.

### Surgical technique

All surgeries were performed by a single surgeon (A.K.) from April, 2011 to December, 2013 at the Department of Ophthalmology, Kanazawa University Graduate School of Medical Science. All patients read and signed an informed consent document prior to enrollment. All DSAEK procedures were performed as previously reported [5]. In brief, donor tissue was dissected with a microkeratome (ALTK Cbm, Moria Japan KK, Tokyo, Japan) equipped with a 300-μm head. After microkeratome dissection, donor tissue was transferred to a punching system and cut with an 8.0 mm diameter punch (Barron donor cornea punch, Katena Products Inc, Denville, NJ). For patients with cataract and endothelial failure (Cases 3 and 4), phacoemulsification and a single-piece acrylic intraocular lens (IOL) insertion procedure was performed from a 3 mm clear corneal temporal incision just prior to DSAEK. This had the benefit of creating more space in the anterior chamber to safely position the graft. Three corneal fenestrations were performed to drain the interface fluid. A small inferior iridectomy at the 6 o’clock position was then created using a 25-gauge vitreous cutter (MIDLAB, San Leandro, CA) under continuous irrigation from a 25-gauge anterior chamber maintainer (Kobayashi 25 g DSAEK Chamber Maintainer, Catalog #AE-7802, ASICO, Westmont, IL) to prevent papillary air block after surgery. Microkeratome-dissected donor tissue was transferred to a punching system and cut with an 8.0 mm diameter punch (Barron donor cornea punch, Katena Products Inc, Denville, NJ). An ophthalmic viscosurgical device (Viscoat; Alcon Laboratories, Fort Worth, TX, USA) was applied to the endothelial surface of the graft, and the donor graft was inserted using a Busin glide and an IOL sheet glide. This is known as the Kobayashi double-glide technique [5]. After insertion of the donor graft, the wound was secured with 3 interrupted 10–0 nylon sutures. Air was injected into the anterior chamber to press the donor graft against the recipient cornea. Corneal massage was performed to adjust the centered position of the donor graft and to eliminate residual fluid at the donor graft-recipient interface. Residual interface fluid was also drained through corneal venting incisions. The anterior chamber was left full of air, and the patients were instructed to lie on their backs for at least 1 hour.

### Visualization of the anterior chamber

**Fig. 2:**
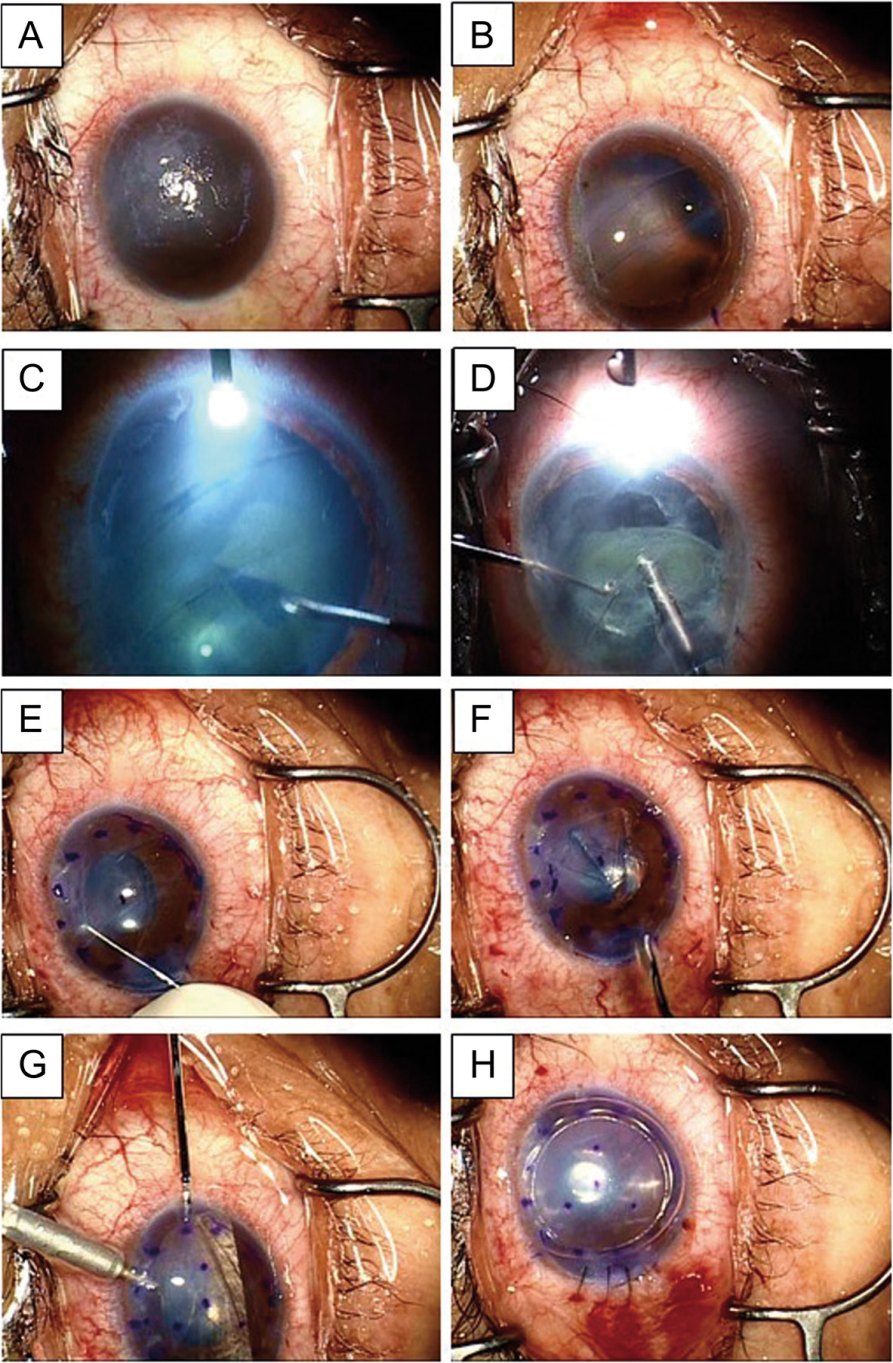
Representative surgical video images of case 3. **a** Before surgery, a severe bullous change prevented complete visualization of the anterior chamber. **b** After removal of the corneal epithelium, visualization of the anterior chamber became possible. **c, d** Continuous curvilinear capsulorhexis and phacoemulsification was possible with the aid of anterior capsule staining by Trypan blue and illumination of the cornea. **e, f** The Descemet’s membrane, 8.0 mm in diameter, with a cord-like lesion was circularly removed. **g** The donor graft, 8.0 mm in diameter, was inserted using a Busin glide and an IOL sheet glide, which is also known as the Kobayashi double-glide technique. **h** After the wound was secured with three interrupted 10–0 nylon sutures, air was injected into the anterior chamber to press the donor graft against the recipient cornea

In cases of difficulties of visualization of the anterior chamber during phacoemulsification and/or DSAEK, corneal epithelial removal, trypan blue staining of the anterior capsule and illumination by an endoillumination probe or surgical slit light were performed for better visualization of the anterior chamber (Fig. 2).

## Results

During DSAEK, all cases required corneal epithelial removal for better visualization of the anterior chamber. Two cases with simultaneous cataract surgeries (cases 3 and 4) required lens anterior capsule staining by trypan blue and illumination of the cornea for further visualization of the anterior chamber (Fig. 2).

**Fig. 3:**
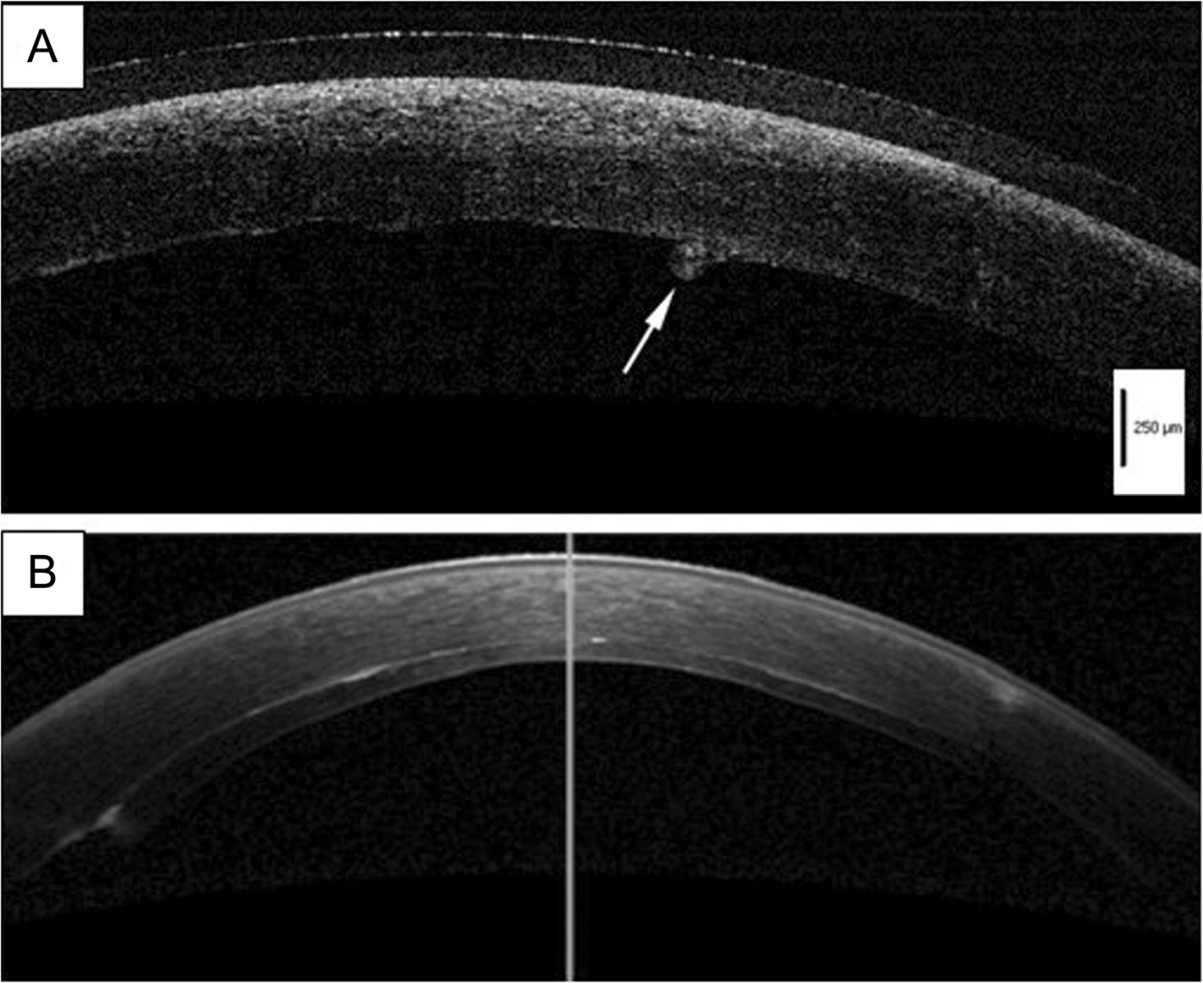
Representative anterior segment optical coherent tomographic images of case 1. **a** Before DSAEK, a thick stroma due to bullous keratopathy with a protrusion of Descemet’s membrane (arrow) was observed. A medical soft contact lens was used in this patient. **b** Six months after DSEAK, the donor endothelial graft was completely attached behind the host cornea. Stromal bullous changes had disappeared

**Fig. 4:**
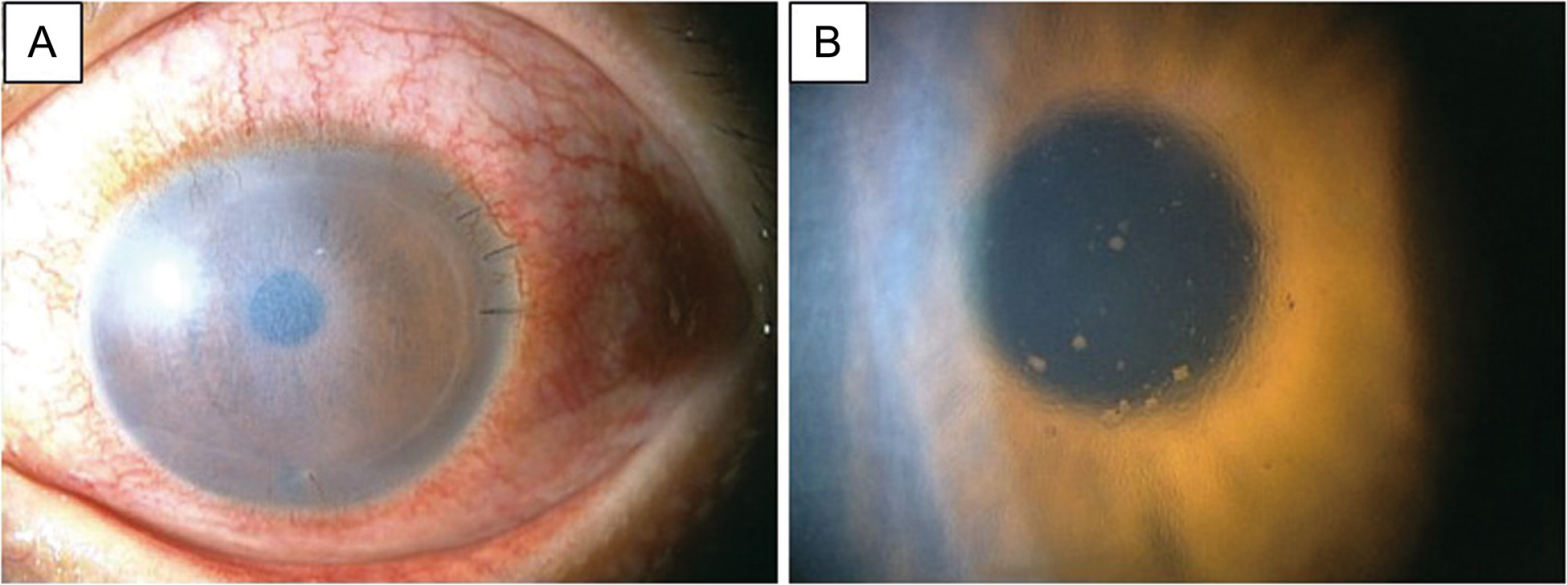
Slit-lamp photo of endothelial rejection in case 4. **a** Four months after DSAEK, endothelial rejection was noted with conjunctival redness. The patient was treated with low dose steroid eye drops. **b** Magnified slit-lamp image of the central cornea. Numerous keratic precipitates were observed

Clinical outcomes of the four patients are summarized in the table (Table 1, Figs. 1 and 3). All patients had a clear graft at the latest follow-up visit. No intraoperative complications were noted. There were no cases of graft dislocation or primary graft failure. In 2 cases, postoperative intraocular pressure (IOP) elevation was noted. In case 3, high IOP (49 mmHg) in the right eye was observed, which was probably due to prolonged use of a steroid eye drop (0.1 % betamethasone). In case 4, high IOP (30 mmHg) was observed the day after surgery. In both cases, IOP reduced rapidly after treatment with oral acetazolamide and latanoprost eye drops. In case 4, a rejection episode was observed at 4 months postoperatively (Fig. 4). A change of eye drop (0.02 % fluorometholone to 0.1 % betamethasone) and an increase in the frequency of eye drop use (3 times per day to 5 times per day) was effective.

In this case series, all patients had limited visual potential due to amblyopia. Mean corrected decimal visual acuity improved from 0.06 to 0.15 at 6 months and to 0.38 at 12 months postoperatively. Postoperative ECD was 2270 ± 415 cells/mm^2^ (mean loss, 24.4 %) at 6 months and 2130 ± 845 (mean loss, 29.1 %) at 12 months. The mean central corneal thickness of the donor cornea was 121.3 ± 24.9 μm (case 1: 128 μm, case 2: 149 μm, case 3: 89 μm, case 4: 119 μm).

## Discussion

Irregular retro-corneal surface is one of the contraindications of DSAEK surgery since perfect smoothness is required for donor host attachment. Endothelial dysfunction due to corneal endothelial injury after forceps injury is rare, but is clinically encountered in cornea clinics [8]. Although direct contact of forceps with the cornea is usually implicated, periocular compression is also a possible mechanism of injury. To date, several case reports of DSAEK for forceps injury have been reported [9–11]. Ponchel *et al*. reported a single case of successful DSAEK for forceps injury for the first time. Subsequently, Haddock *et al*. also reported a single case of successful DSAEK for forceps injury. Recently, Hayashi *et al*. reported 6 cases of DSAEK for irregular corneal posterior surface in which 5 cases were due to birth injuries [11]. They also reported that all five DSAEK were successful without notable complications. However, all cases showed no preoperative stromal opacity [9–11].

In this report, we confirmed the usefulness of DSAEK for an irregular corneal posterior surface due to forceps injury. Stripping of the posterior Descemet’s membrane to make the posterior surface smooth was proven possible in all of our cases, even in patients with a mean age of 51.5 years. Most notably, even though the corneal stroma had severe bullous changes as reported herein, DSAEK and concomitant phacoemulsification was possible using several surgical techniques to enhance visualization of the anterior chamber. For DSAEK only (cases 1 and 2), epithelial removal improved visualization of the anterior chamber enough to perform DASEK. For DSAEK with simultaneous cataract surgery (cases 3 and 4), further techniques were necessary to obtain enough visibility for safe surgeries including trypan blue staining of the lens anterior capsule and illumination with an endoillumination probe (case 3, Fig. 2) or by a surgical slit lamp (case 4). These visualization techniques are already reported [5, 12, 13]. However, it should be noted that the combination of these techniques is quite useful not only for enhancement of visualization of cataract surgery, but also for irregular Descemet’s membrane removal through bullous stromal changes. In addition, the combination of these visualization techniques is also useful for Descemet’s membrane endothelial keratoplasty through a hazy cornea with severe stromal bullous changes [14].

In this case series, no intraoperative complications were noted. However, two cases of IOP elevation were observed. One case (case 3) was due to prolonged use of betamethasone eye drops for a putative steroid responder and the other case (case 4) was a transient postoperative IOP elevation. In both cases, IOP reduced rapidly after oral acetazolamide treatment. Although the causative reason was unclear, an endothelial rejection episode was observed in case 4 at 4 months postoperatively (Fig. 4). Collectively, as is the case with uncomplicated DSAEK, meticulous care is required during the follow-up of DSAEK after birth injuries.

## Conclusions

In conclusion, the clinical outcome of DSAEK for severe bullous keratopathy after forceps delivery was fair with rapid corneal clearance, which was comparable to uncomplicated cases. Cataract and DSAEK surgery was safely performed using several techniques such as epithelial removal, lens anterior capsule staining and illumination of the cornea. These techniques enabled better visualization of the anterior chamber. Although all patients had improved postoperative visual acuity, it was limited due to preexisting amblyopia. Meticulous care was required for postoperative complications including intraocular pressure rise and endothelial rejection.

## Abbreviations

DSAEK: Descemet’s stripping automated endothelial keratoplasty
ECDs: Endothelial-cell densities
